# PrEP surfing: HIV preexposure prophylaxis use of sex partners as HIV prevention strategy among men who have sex with men

**DOI:** 10.1097/QAD.0000000000004526

**Published:** 2026-04-21

**Authors:** Carien M. Blomaard, Jeffrey C.D. Koole, Elske Hoornenborg, Maria Prins, Janneke C.M. Heijne, Udi Davidovich, Maarten F. Schim Van Der Loeff

**Affiliations:** a Department of Infectious Diseases, Public Health Service Amsterdam; b Department of Internal Medicine, Amsterdam UMC location University of Amsterdam, Amsterdam, The Netherlands; c Amsterdam Institute for Immunology and Infectious Diseases (AII); d Amsterdam Public Health Research Institute (APH); e Department of Social Psychology, University of Amsterdam, Amsterdam, The Netherlands

**Keywords:** HIV prevention & control, men who have sex with men, preexposure prophylaxis, sexual behavior

## Abstract

**Objective:**

Having condomless anal sex while relying on the HIV preexposure prophylaxis (PrEP) use of one's sex partner is referred to as PrEP surfing. We aimed to estimate the proportion of men who have sex with men (MSM) practicing PrEP surfing, their intention to practice it, and determinants of both.

**Design:**

Open prospective cohort study.

**Methods:**

We included MSM participating in the Amsterdam Cohort Studies in 2021–2023. Biannually, participants completed questionnaires on recent (=preceding 6 months) sexual behavior, including PrEP use, PrEP surfing, and intention for both in the coming 6 months (7-point Likert scale). We assessed determinants of PrEP surfing with logistic regression and determinants of intention with linear regression.

**Results:**

We included 2187 visits from 636 participants to analyze intention for PrEP surfing. Median age was 45 [interquartile range (IQR) = 34–52] years, median number of recent sex partners 4 (IQR = 1–11), and 264 (41%) participants reported recent PrEP use. For recent PrEP surfing, 1779 visits from 562/636 (88%) participants with event-driven/no PrEP use were analyzed. Recent PrEP surfing was reported in 449/1779 (25%) visits. Median intention for PrEP surfing was 4 (IQR = 1–6); in 588/2187 (27%) visits, intention for PrEP surfing was high (=score 6–7). Recent PrEP use and high perceived probability of acquiring HIV were associated with recent PrEP surfing and higher intention, while age ≥45 and having a steady partner were inversely associated. Having ≥3 recent partners was associated with recent PrEP surfing.

**Conclusions:**

PrEP surfing is a common HIV prevention strategy among MSM in Amsterdam. Its effectiveness needs to be determined to optimize HIV prevention care.

## Introduction

Men who have sex with men (MSM) are at increased risk for acquiring HIV. Daily and event-driven oral preexposure prophylaxis (PrEP) with tenofovir/emtricitabine (TDF/FTC) are highly effective in preventing HIV acquisition [1,2]. However, not all PrEP eligible MSM are using it because of barriers in awareness, motivation, access or effective use [3–7]. First, eligible MSM may not perceive themselves at increased risk for HIV [8,9]. Second, some do not want to use PrEP (e.g., because of the burden of daily pill-taking) [6]. Third, those willing to use PrEP may have difficulties accessing it (e.g., because of costs or limited PrEP program capacities) [10,11]. Last, those who accessed PrEP may use it incorrectly or discontinue using it (the latter because of e.g., side-effects, running out of pill supply) [12]. MSM also apply other HIV prevention strategies, such as condoms or viral load sorting (undetectable equals untransmittable [U = U]) [13–15].

The growing use of PrEP among MSM has resulted in the emergence of a new HIV prevention strategy: *PrEP surfing* (Jonas, personal communication, September 2024). This refers to having condomless anal sex (CAS) while relying on the HIV PrEP use of one's sex partner. Like U = U, PrEP surfing is an indirect method of HIV prevention, which relies on the sex partner's use of medication [16]. However, it is unknown how often PrEP surfing is practiced, who practices it, and to what extent MSM intend to practice it. Insight in both intention and actual behavior of PrEP surfing is essential to assess the public health impact of this emerging strategy of HIV prevention, and to inform sexual healthcare [16].

Here, we aim to assess the proportion of HIV-negative MSM practicing PrEP surfing with casual partners, determinants of practicing PrEP surfing, the intention to practice PrEP surfing and its determinants, and to which degree the intention to practice PrEP surfing is associated with actual practice.

## Methods

### Study design and participants

The Amsterdam Cohort Studies (ACS) is an open prospective cohort study, initiated in 1984 [17]. The aim of ACS is to study the epidemiology, determinants, pathogenesis, and course of HIV-1 infection, sexually transmitted infections (STI) and blood-borne infections other than HIV. Men aged 16 years and older were eligible for participation if they reported sex with other men in the preceding six months and live in/around Amsterdam, the Netherlands, or frequently participate in MSM-related activities in this area [18]. ACS participants attend face-to-face semi-annual study visits at the Public Health Service of Amsterdam. Prior to these visits, participants complete an online questionnaire on health, sexual behavior (e.g., number of sex partners, sexualized drug use) and HIV prevention strategies (e.g., PrEP use, condom use, viral load sorting). From October 2021 onwards, data on PrEP surfing were collected among participants who reported anal sex with steady or casual partners in the preceding six months.

During study visits, samples are taken for testing on HIV and other STIs according to the protocol of the Centre for Sexual Health Amsterdam. Sociodemographic characteristics were collected at enrolment. The ACS is approved by the Medical Ethics Review Board of the Amsterdam University Medical Center, the Netherlands (2007-182-NL18679.018.07-A2007-182.0001). Participation is voluntary and each participant provides written informed consent before enrolment.

In the Netherlands, PrEP can be accessed formally through Public Health Services or general practitioners [19]. In ACS, PrEP is prescribed to eligible participants requesting it since 2019.

### Data collection and variables

HIV-negative ACS participants with study visits between October 2021 and December 2023 were included in the analysis of intention for PrEP surfing. Men were eligible for analysis of recently practiced PrEP surfing if they reported event-driven PrEP use (with or without periods of daily PrEP use) or reported no PrEP use; those who reported PrEP use and indicated they had only used PrEP on a daily regimen in the preceding six months were excluded from this analysis. PrEP surfing was defined as using one's partner's PrEP use as HIV prevention strategy when engaging in CAS with that partner. After providing a short explanation of PrEP surfing, participants were asked if they had practiced PrEP surfing with casual sex partners in the previous six months. They were also asked to score their intention to practice PrEP surfing with HIV-negative sex partners in the coming 6 months on a 7-point Likert scale (see Supplementary Methods, Supplemental Digital Content). High intention to practice PrEP surfing was defined as ≥6, intermediate intention as 3–5 and low intention as ≤2.

Intention to use oral PrEP oneself in the coming six months was scored on a 7-point Likert scale. PrEP eligibility of participants was assessed based on then current Dutch PrEP guidelines (i.e., CAS with a partner with unknown HIV status or unknown or detectable viral load, rectal STI or infectious syphilis, or postexposure prophylaxis (PEP) use, all in the previous 6 months) [19]. Sexualized drug use included substance use before/during sex, excluding alcohol, marijuana, poppers and erection stimulants use, following conventions [20]. HIV acquisition perceptions, including perceived probability of contracting HIV, worrying about contracting HIV, importance of preventing HIV acquisition, and perceived burden of acquiring HIV, all in the coming 6 months, were measured using a 7-point Likert scale.

### Statistical analysis

Descriptive data were expressed as number and percentage, mean [standard deviation, (SD)] or median (interquartile range, IQR) depending on their distribution. The primary outcomes were: having practiced PrEP surfing in the preceding six months (henceforth referred to as ‘recent PrEP surfing’), and intention to practice PrEP surfing in the coming 6 months.

PrEP surfing has no objective prevention benefit for MSM who consistently use daily PrEP. Therefore, we excluded visits of men who reported PrEP use only on a daily regimen from the analysis of determinants of recent PrEP surfing. Logistic regression was applied, with standard errors adjusting for repeated measurements of individuals. In the analysis of determinants for intention to practice PrEP surfing, all visits were included, also those of daily PrEP users, as recent daily PrEP use does not guarantee daily use in the coming months. We used linear regression with intention as outcome, with standard errors adjusting for repeated measurements of individuals. For both analyses we used the following approach. We first performed univariable analyses including variables on sociodemographic characteristics, sexual behavior, HIV PrEP characteristics and HIV risk perception. Next, we performed multivariable analysis including variables with *P* < 0.20 from the univariable analysis and using backward selection based on likelihood ratios to identify determinants significantly associated with the outcome. We did not include ‘PrEP eligibility’ as covariate in the multivariable model because of the expected multicollinearity with various other variables. The variables ‘CAS with a partner with unknown HIV status’ and ‘CAS with a partner with HIV with an unknown/detectable viral load’ were also excluded because of expected multicollinearity with the variable ‘CAS with a casual partner’. Finally, the variables ‘sex work’, ‘injecting drug use’ and ‘PEP use’ were excluded from multivariable models because of very small numbers (≤15) of participants reporting these practices.

Missing data for HIV risk perception variables (worried about contracting HIV, burden of contracting HIV, and importance of preventing HIV acquisition) occurred because in one semi-annual study round the corresponding questions were not posed (October 2022 to April 2023). We evaluated whether HIV perceptions fluctuated over time among our participants. As the median difference between values reported by participants for each HIV perception variable between subsequent visits was 0 (IQR 0–0), we imputed the missing data (462 records) using the average of a participant's preceding and subsequent visit. HIV risk perception variables were highly skewed, with very small numbers at one extreme (Supplementary Figure 1), so we dichotomized these variables in logistic regression analyses.

To assess to which degree the intention for PrEP surfing predicts practicing it, we assessed the association between the actual practice of PrEP surfing in the preceding 6 months (reported at visit *t* = *n*), and intention of practicing PrEP surfing in the coming six months (reported at visit *t* = *n* − 1). We excluded baseline visits (i.e., first study visit in study period) from this analysis, as these lacked a preceding visit with reported intention for PrEP surfing. We used logistic regression with standard errors adjusting for correlated observations, with PrEP surfing as outcome and intention to practice it as explanatory variable. Variables that were associated with recent PrEP surfing in the multivariable analysis of determinants for recent PrEP surfing were included in the multivariable model.

All analyses were carried out using STATA software (v17.0, StataCorp, College Station, TX, USA). A two-sided *P*-value <0.05 was considered statistically significant.

## Results

### Sociodemographics

Between October 2021 and December 2023, 2348 visits by 646 HIV-negative MSM took place, with 161 (6.9%) visits missing PrEP surfing data because the questionnaire was not completed. 636 HIV-negative MSM participating in ACS provided data on PrEP surfing in 2187 visits. The median age was 45 (IQR 34–52) years (Table 1). Most participants were born in the Netherlands (*n* = 519, 85.9%), had a college degree (*n* = 517, 81.3%), and were employed (*n* = 499, 79.6%). Most participants had a steady partner (*n* = 499, (79.6%). The median number of sex partners in the preceding 6 months was 4 (IQR 1–11). CAS with a casual partner was reported by 318 (50.0%) participants. 232 (36.5%) participants were PrEP eligible in the preceding 6 months, and 264 (41.5%) had used PrEP, of whom 99 (37.5%) used daily PrEP.

**Table 1:** Sociodemographic, sexual behavior and PrEP surfing characteristics at baseline of 636 HIV-negative men who have sex with men in the Amsterdam Cohort Studies, the Netherlands, 2021–2023.

	Total (*N* = 636)
	
	median (IQR), *n* (%)
*Sociodemographic characteristics*	
Age in years	45 (34–52)
Age in years, categories	
16–34	167 (26.3%)
35–44	144 (22.6%)
45–54	209 (32.9%)
≥55	116 (18.2%)
Residence	
Amsterdam	574 (90.3%)
Other	62 (9.7%)
Country of birth	
The Netherlands	519 (85.9%)
Other	85 (14.1%)
Educational level	
None/primary/(post)secondary	119 (18.7%)
College/university	517 (81.3%)
Employed	
Yes	499 (79.6%)
No	128 (20.4%)
*Sexual behavior in preceding 6 months*	
Steady partner	
Yes	499 (79.6%)
No	128 (20.4%)
Sex work	
No	635 (99.8%)
Yes	1 (0.2%)
Number of sex partners	4 (1–11)
Number of partners, tertiles	
0–2	241 (37.9%)
3–8	208 (32.7%)
≥9	187 (29.4%)
Sexualized drug use^a^	
No	421 (66.2%)
Yes	215 (33.8%)
Injecting drug use	
No	632 (99.4%)
Yes	4 (0.6%)
Group sex	
No	441 (69.3%)
Yes	195 (30.7%)
Condomless anal sex with steady partner	
No	377 (59.3%)
Yes	259 (40.7%)
Condomless anal sex with casual partner	
No	318 (50.0%)
Yes	318 (50.0%)
Condomless anal sex with partner with unknown HIV status	
No	440 (69.2%)
Yes	196 (30.8%)
Condomless anal sex with partner with HIV and unknown/detectable viral load	
No	604 (95.0%)
Yes	32 (5.0%)
Rectal STI or infectious syphilis	
No	573 (90.1%)
Yes	63 (9.9%)
HIV PEP use	
No	635 (99.8%)
Yes	1 (0.2%)
*PrEP characteristics in preceding 6 months*	
PrEP eligible^b^	
No	404 (63.5%)
Yes	232 (36.5%)
PrEP use^c^	
No	372 (58.5%)
Yes	264 (41.5%)
PrEP regimen^d^	
Daily	99 (37.5%)
Event-driven	124 (47.0%)
Both daily and event-driven	41 (15.5%)
*HIV risk perception and attitudes in coming 6 months* ^e^	
Worried about contracting HIV (1–7)	2.0 (1.0–4.0)
Worried about contracting HIV	
Low (1–2)	378 (62.6%)
Intermediate (3–5)	175 (29.0%)
High (6–7)	51 (8.4%)
Probability of contracting HIV (1–7)	2.0 (1.0–2.0)
Probability of contracting HIV	
Low (1–2)	531 (83.5%)
Intermediate (3–5)	103 (16.2%)
High (6–7)	2 (0.3%)
Burden of contracting HIV (1–7)	7.0 (6.0–7.0)
Burden of contracting HIV	
Low (1–2)	9 (1.5%)
Intermediate (3–5)	48 (7.9%)
High (6–7)	547 (90.6%)
Importance of preventing HIV acquisition (1–7)	7.0 (7.0–7.0)
Importance of preventing HIV acquisition	
Low (1–2)	7 (1.2%)
Intermediate (3–5)	40 (6.6%)
High (6–7)	557 (92.2%)
*PrEP surfing*	
PrEP surfing in preceding 6 months	
No	452 (71.1%)
Yes	184 (28.9%)
Intention to practice PrEP surfing in coming 6 months^f^	4.0 (2.0–6.0)
Intention to practice PrEP surfing in coming 6 months^f^	
Low (1–2)	233 (36.6%)
Intermediate (3–5)	233 (36.6%)
High (6–7)	170 (26.7%)

%, percentage; IQR, interquartile range; *n*, number; *N*, total number; PEP, HIV postexposure prophylaxis; PrEP, oral HIV preexposure prophylaxis; SD, standard deviation.

Data were missing for: country of birth (*n* = 32), employment status (*n* = 9), worried about contracting HIV (*n* = 32), burden of contracting HIV (*n* = 32), and importance of preventing HIV acquisition (*n* = 32).

a Sexualized drug use included any drug use before or during sex, excluding alcohol, poppers, erectile stimulants and marijuana use.

b PrEP eligible was defined as having at least one of the following in the preceding six months: condomless anal sex with a male partner with unknown HIV status, or with a partner with HIV with either unknown or detectable viral load; rectal STI or infectious syphilis; or PEP use.

c At baseline, 264 participants were using PrEP while 232 were PrEP eligible. Since PrEP eligibility can fluctuate over time, also among PrEP users, some participants may have used PrEP in the preceding 6 months despite not meeting the eligibility criteria at that time, though they did meet these criteria in the past.

d Among participants reporting PrEP use in preceding 6 months.

e HIV risk perception and attitudes were measured using a 7-point Likert scale, with a score of ≥6 defined as high.

f Intention to practice PrEP surfing was measured using a 7-point Likert scale, with a score of ≥6 defined as high.

### Recently practiced PrEP surfing

**Fig. 1:**
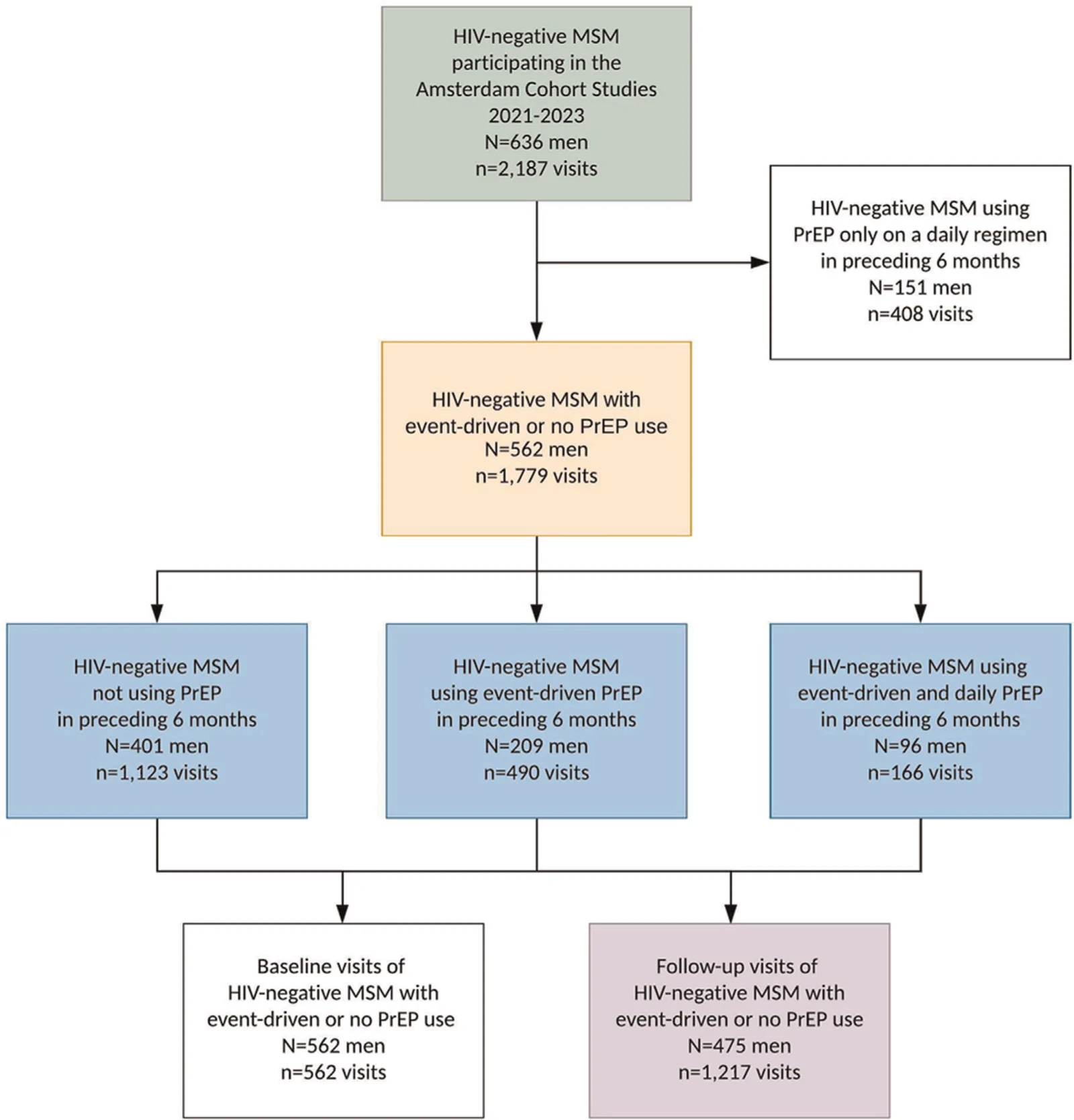
Flowchart of men who have sex with men included in analyses on HIV preexposure prophylaxis (PrEP) surfing, participating from October 2021–December 2023 in the Amsterdam Cohort Studies, the Netherlands.

Of 636 participants with data on PrEP surfing, 562 (88.4%) participants used event-driven or no PrEP during the study period and were included in the analysis of practiced PrEP surfing. The 562 participants contributed 1779 visits (Fig. 1). Recent PrEP surfing was reported at least once by 238 (42.4%) participants and recorded in 449 (25.2%) of all study visits. In multivariable analysis, age ≥45 years was negatively associated with recently practiced PrEP surfing [45–54 years: adjusted odds ratio (aOR) 0.5, 95% confidence interval (CI) 0.3–0.7; and ≥55 years: aOR 0.5, 95% CI 0.3–0.7] compared to age 18–34 years (Table 2). A country of origin other than the Netherlands was negatively associated with recent PrEP surfing (aOR 0.6, 95% CI 0.3–1.0). More participants with 3–8 sex partners (aOR 4.1, 95% CI 2.8–5.9) or ≥9 sex partners (aOR 4.1, 95% CI 2.7–6.4) in the preceding 6 months reported PrEP surfing than participants with 0–2 sex partners. Participants who reported recent event-driven PrEP use had higher odds of recently practiced PrEP surfing (aOR 3.1, 95% CI 2.2–4.3), as did participants with diagnosed rectal STI or syphilis in the preceding 6 months (aOR 1.6, 95%CI 1.1–2.4). Participants without a steady partner were more likely to report recent PrEP surfing than those with a steady partner (aOR 1.5, 95% CI 1.1–2.1). Participants who perceived their probability of contracting HIV as intermediate/high (aOR 1.5, 95% CI 1.1–2.2) reported recent PrEP surfing more often than participants with low HIV acquisition probability perception.

**Table 2:** Determinants of practiced PrEP surfing in preceding 6 months in 1779 study visits among 562 HIV-negative men who have sex with men with event-driven or no PrEP use, participating in the Amsterdam Cohort Studies, the Netherlands, 2021–2023.

		Univariable model		Multivariable model		
	*n*/*N* (%)	OR	95% CI	aOR	95% CI	*P*
Age group						
16–34 years	135/405 (33.3%)	REF	–	REF	–	–
35–44 years	117/363 (32.2%)	0.95	0.63–1.42	1.03	0.67–1.58	0.900
45–54 years	115/612 (18.8%)	0.46	0.31–0.68	0.48	0.32–0.73	<0.001
≥55 years	82/399 (20.6%)	0.52	0.33–0.81	0.47	0.29–0.73	<0.001
Residence						
Amsterdam	411/1634 (25.2%)	REF	–			
Other	38/145 (26.2%)	1.06	0.64–1.75			
Country of origin						
The Netherlands	393/1504 (26.1%)	REF	–	REF	–	–
Other	41/239 (17.2%)	0.59	0.37–0.93	0.57	0.34–0.96	0.036
Educational level						
None/primary/(post) secondary	64/301 (21.3%)	REF	–			
College/university	385/1478 (26.1%)	1.30	0.86–1.97			
Employment						
Yes	358/1408 (25.4%)	REF	–			
No	85/342 (24.9%)	0.97	0.66–1.43			
Steady partner^a^						
Yes	261/1215 (21.5%)	REF	–	REF	–	–
No	188/564 (33.3%)	1.83	1.37–2.44	1.51	1.10–2.08	0.010
Sex work^a^						
No	448/1777 (25.2%)	N.A.				
Yes	1/2 (50.0%)	N.A.				
Number of sex partners^a^						
0–2	64/745 (8.6%)	REF	–	REF	–	–
3–8	204/577 (35.4%)	5.82	4.06–8.34	4.09	2.82–5.94	<0.001
≥9	181/457 (39.6%)	6.98	4.67–10.42	4.11	2.66–6.35	<0.001
Group sex^a^						
No	259/1272 (20.4%)	REF	–			
Yes	190/507 (37.5%)	2.34	1.79–3.07			
CAS with steady partner^a^						
No	266/1025 (26.0%)	REF	–			
Yes	183/754 (24.3%)	0.91	0.69–1.21			
CAS with casual partner^a^						
No	60/1004 (6.0%)	REF	–	N.A.		
Yes	389/775 (50.2%)	15.86	11.34–22.17	N.A.		
CAS with partner with unknown HIV status^a^						
No	243/1292 (18.8%)	REF	–			
Yes	206/487 (42.3%)	3.16	2.39–4.23			
CAS with partner with unknown/detectable viral load^a^						
No	430/1727 (24.9%)	REF	–			
Yes	19/52 (36.5%)	1.74	0.84–3.59			
Sexualized drug use^a^^,^^b^						
No	243/1223 (19.9%)	REF	–			
Yes	206/556 (37.1%)	2.37	1.77–3.18			
Injecting drug use^a^						
No	446/1772 (25.2%)	N.A.				
Yes	3/7 (42.9%)	N.A.				
Rectal STI or syphilis^a^						
No	367/1610 (22.8%)	REF	–	REF	–	–
Yes	82/169 (48.5%)	3.19	2.24–4.55	1.61	1.09–2.37	0.016
PEP use^a^						
No	449/1774 (25.3%)	N.A.				
Yes	0/5 (0.0%)	N.A.				
PrEP eligible^a^^,^^c^						
No	206/1205 (17.1%)	REF	–	N.A.		
Yes	243/574 (42.3%)	3.56	2.69–4.71	N.A.		
PrEP use^a^^,^^d^						
No	164/1123 (14.6%)	REF	–	REF	–	–
Yes	285/656 (43.5%)	4.49	3.37–5.99	3.09	2.23–4.29	<0.001
Worried about contracting HIV^e^						
Low (1–2.5)	221/1028 (21.5%)	REF	–			
Intermediate/high (3–7)	225/747 (30.1%)	1.57	1.20–2.06			
Perceived probability of contracting HIV^e^						
Low (1–2.5)	355/1500 (23.7%)	REF	–	REF	–	–
Intermediate/high (3–7)	94/279 (33.7%)	1.64	1.21–2.22	1.53	1.07–2.20	0.019
Importance of preventing HIV acquisition^e^						
Low/intermediate (1–5)	30/117 (25.6%)	REF	–			
High (5.5–7)	416/1658 (25.1%)	0.97	0.60–1.57			
Expected burden of contracting HIV^e^						
Low/intermediate (1–5)	28/129 (21.7%)	REF	–			
High (5.5–7)	418/1646 (25.4%)	1.22	0.75–2.02			

Results from logistic regression with standard errors adjusting for correlated observations.

aOR, adjusted odds ratio; CAS, condomless anal sex; CI, confidence interval; HIV, human immunodeficiency virus; IQR, interquartile range; *n*, number; *N*, total number; N.A., not applicable; OR, odds ratio; *P*, *P*-value; PEP, oral HIV postexposure prophylaxis; PrEP, oral HIV preexposure prophylaxis; STI, sexually transmitted infection.

Data were missing for: country of origin (*n* = 36), worried about contracting HIV (*n* = 4, after imputation), importance of preventing HIV (*n* = 4, after imputation) and burden of contracting HIV (*n* = 4, after imputation).

a In preceding 6 months.

b Sexualized drug use included any drug use before or during sex, excluding alcohol, poppers, erectile stimulants and marihuana use.

c PrEP eligible was defined as having at least one of the following in the preceding six months: condomless anal sex with a male partner with unknown HIV status, or with a partner with HIV with either unknown or detectable viral load; rectal STI or infectious syphilis; or PEP use.

d Event-driven or both daily and event-driven PrEP use. Participants using PrEP only on a daily regimen in the preceding 6 months were excluded from this analysis.

e In coming 6 months.

### Intention to practice PrEP surfing

636 participants contributed 2187 study visits (Fig. 1). The median intention to practice PrEP surfing was 4 (IQR 1–6). Having a high intention (score 6–7) to practice PrEP surfing was recorded in 588/2187 (26.9%) study visits. Of these 636 participants, 306 (48.1%) reported high intention to practice PrEP surfing during at least one of their study visits. 192 (30.2%) participants reported medium intention (score 3–5) and 138 (21.7%) low intention (score 1–2) to practice PrEP surfing in all their study visits. In multivariable analysis, we found that CAS with casual partners in the preceding 6 months (*β* = 1.1, 95% CI 0.8–1.4), being intermediate/highly worried about contracting HIV (*β* = 0.3, 95% CI 0.0–0.5), and perceiving one's own probability of contracting HIV as intermediate/high (*β* = 0.5, 95% CI 0.2–0.8) were positively associated with higher intention for PrEP surfing in the coming 6 months (Table 3). PrEP use in the preceding 6 months was associated with intention for PrEP surfing (*β* = 0.7, 95% CI 0.3–1.2). Number of sex partners was not associated with intention for PrEP surfing, but an interaction between the number of sex partners and PrEP use was observed. Specifically, men reporting PrEP use and ≥9 sex partners had lower intention to PrEP surfing than those reporting 0–2 sex partners and no PrEP use. Being aged ≥45 years was negatively associated with intention for PrEP surfing (45–54 years; *β* = −0.8, 95% CI −1.2, −0.5 and ≥55 years; *β* = −0.7, 95% CI −1.1, −0.3), compared to an age of 18–34 years. Participants without a steady partner had higher intention to practice PrEP surfing than those with a steady partner (*β* = 0.4, 95% CI 0.1–0.7).

All 2187 visits (contributed by 636 men) which took place between October 2021 and December 2023 were included in the analysis of intention for PrEP surfing and determinants of intention. 408 visits (contributed by 151 men) were excluded from analyses into recently practiced PrEP surfing because participants reported PrEP use only on a daily regimen, leaving 1779 visits (contributed by 562 men) for the analysis of proportion and determinants of recently practiced PrEP surfing. For the analysis of intention-behaviour agreement, we excluded all 562 baseline visits since for those there was no preceding visit with reported intention to PrEP surfing.

**Table 3:** Determinants of intention to practice PrEP surfing^a^ in 2187 visits among 636 HIV-negative men who have sex with men participating in the Amsterdam Cohort Studies, the Netherlands, 2021–2023.

		Univariable model		Multivariable model		
	Median (IQR)	coefficient	95% CI	coefficient	95% CI	*P*
Age group						
16–34 years	5 (2–6)	REF	–	REF	–	–
35–44 years	4 (2–6)	−0.27	−0.67, 0.13	−0.17	−0.53, 0.18	0.341
45–54 years	3 (1–5)	−0.96	−1.33, −0.60	−0.83	−1.15, −0.50	<0.001
≥55 years	3 (1–5)	−0.79	−1.21, −0.36	−0.68	−1.07, −0.29	0.001
Residence						
Amsterdam	4 (1–6)	REF	–			
Not in Amsterdam	5 (2–6)	0.56	0.05, 1.07			
Country of origin						
The Netherlands	4 (2–6)	REF	–			
Other	4 (1–5)	−0.22	−0.64, 0.20			
Educational level						
None/primary/secondary/(post)secondary	4 (1–6)	REF	–			
College/university	4 (2–6)	−0.01	−0.40, 0.38			
Employment						
Yes	4 (1–6)	REF	–			
No	4 (2–6)	0.18	−0.19, 0.55			
Steady partner^b^						
Yes	3 (1–5)	REF	–	REF	−	
No	5 (2–6)	0.73	0.45, 1.01	0.40	0.14, 0.66	0.003
Sex work^b^						
No	4 (1–6)	N.A.				
Yes	6.5 (6–7)	N.A.				
Number of sex partners^b^						
0–2	3 (1–5)	REF	–	REF	–	
3–8	4 (2–6)	0.65	0.37, 0.93	−0.21	−0.55, 0.13	0.224
≥9	4 (2–6)	0.36	0.05, 0.68	−0.27	−0.75, 0.21	0.272
Number of sex partners x PrEP use^b^^,^^c^						
0–2 (PrEP use)	5 (2–6)	REF	–	REF	–	
3–8 (PrEP use)	5 (2–6)	−0.14	−0.70, 0.41	−0.06	−0.58, 0.46	0.813
≥9 (PrEP use)	4 (2–6)	−0.86	−1.54, −0.18	−0.77	−1.39, −0.15	0.014
Group sex^b^						
No	4 (1–6)	REF	–			
Yes	4 (2–6)	0.16	−0.11, 0.42			
CAS with steady partner^b^						
No	4 (2–6)	REF	–			
Yes	3 (1–5)	−0.37	−0.63, −0.10			
CAS with casual partner^b^						
No	2 (1–5)	REF	–	REF	–	–
Yes	5 (2–6)	1.07	0.82, 1.32	1.05	0.75, 1.35	<0.001
CAS with partner with unknown HIV status^b^						
No	4 (1–6)	REF	–			
Yes	4 (2–6)	0.28	0.02, 0.54			
CAS with partner with unknown/detectable viral load^b^						
No	4 (2–6)	REF	–			
Yes	2 (1–4)	−0.72	−1.17, −0.28			
Sexualized drug use^b^^,^^d^						
No	4 (1–5)	REF	–			
Yes	4 (2–6)	0.42	0.14, 1.70			
Injecting drug use^b^						
No	4 (1–6)	N.A.				
Yes	4 (3–6)	N.A.				
Rectal STI or syphilis^b^						
No	4 (1–6)	REF	–			
Yes	4 (2–6)	0.40	0.07, 0.72			
PEP use^b^						
No	4 (1–6)	N.A.				
Yes	3 (2–4)	N.A.				
PrEP eligible^b^^,^^e^						
No	4 (1–5)	REF	–			
Yes	4 (2–6)	0.32	0.07, 0.57			
PrEP use^b^						
No	3 (1–5)	REF		REF	–	–
Yes	4 (2–6)	0.86	0.60, 1.13	0.73	0.30, 1.16	0.001
Worrying about contracting HIV^f^						
Low (1–2.5)	3 (1–5)	REF	–	REF	–	–
Intermediate/high (3–7)	4 (2–6)	0.55	0.30, 0.79	0.26	0.03, 0.50	0.026
Perceived probability of contracting HIV^f^						
Low (1–2.5)	4 (1–6)	REF	–	REF	–	
Intermediate/high (3–7)	5 (3–6)	0.79	0.52, 1.06	0.49	0.24, 0.75	< 0.001
Importance of preventing HIV acquisition^f^						
Low/intermediate (1–5)	4 (2–5)	REF	–			
High (5.5–7)	4 (1–6)	0.19	−0.19, 0.57			
Burden of contracting HIV^f^						
Low/intermediate (1–5)	3 (1–5)	REF	–			
High (5.5–7)	4 (1–6)	0.63	0.28, 0.99			

Results from linear regression with standard errors adjusting for correlated observations.

CAS, condomless anal sex; CI, confidence interval; HIV, human immunodeficiency virus; IQR, interquartile range; *n*, number; *N*, total number; OR, odds ratio; *P*, *P*-value; PEP, oral HIV postexposure prophylaxis; PrEP, oral HIV preexposure prophylaxis; STI, sexually transmitted infection.

Data were missing for: country of origin (*n* = 45), employment (*n* = 29), worried about acquiring HIV (*n* = 5, after imputation), importance of preventing HIV (*n* = 5, after imputation) and burden of contracting HIV (*n* = 5, after imputation).

a Intention to practice PrEP surfing was measured using a 7-point Likert scale.

b In preceding 6 months.

c The interaction term indicates how the number of sex partners and PrEP use interact with each other on the outcome.

d Sexualized drug use included any drug use before or during sex, excluding alcohol, poppers, erectile stimulants and marihuana use.

e PrEP eligible was defined as having at least one of the following in the preceding six months: condomless anal sex with a male partner with unknown HIV status, or with a partner with HIV with either unknown or detectable viral load; rectal STI or infectious syphilis; or PEP use.

f In coming 6 months.

### Intention as predictor for practice

**Fig. 2:**
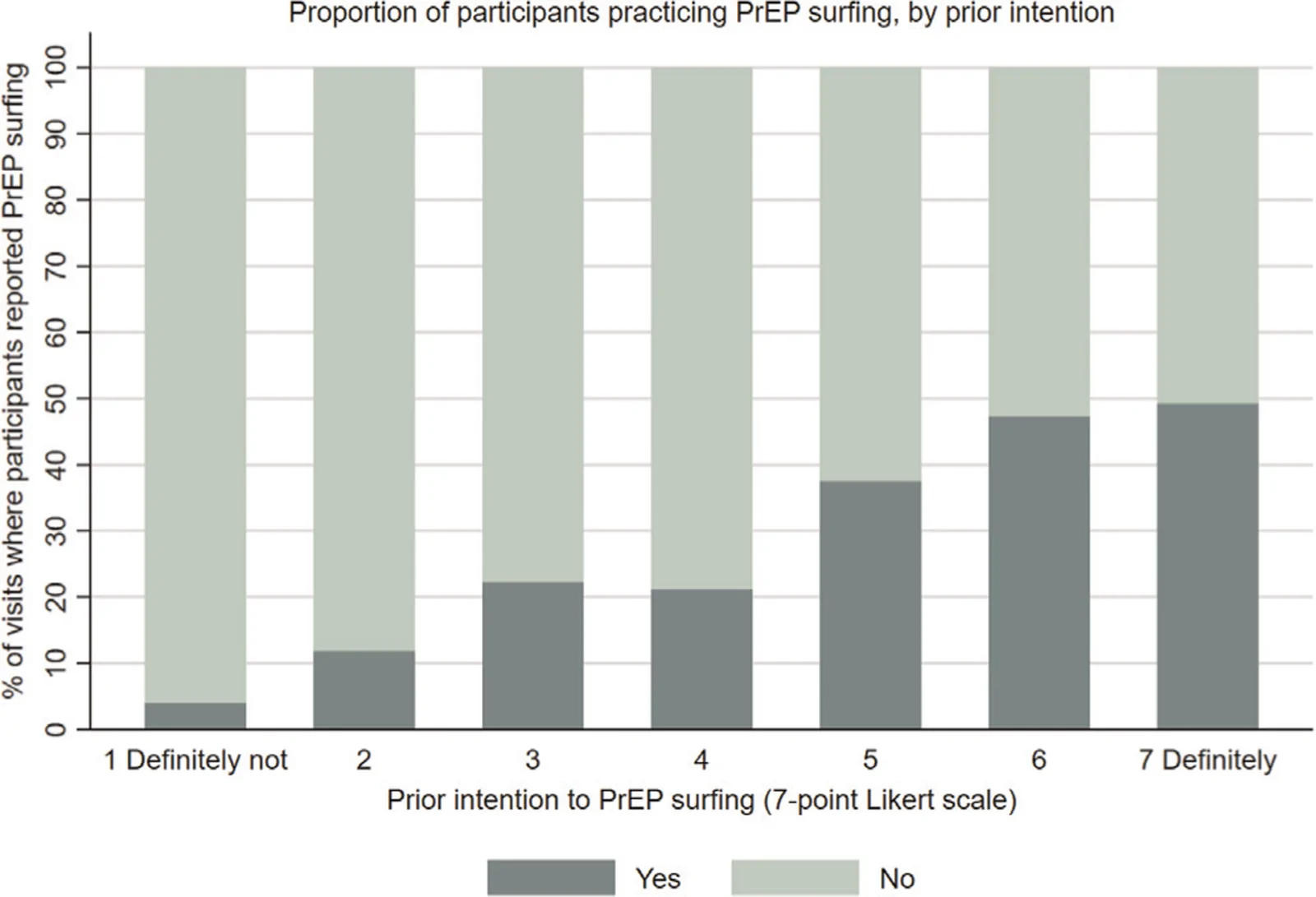
Proportion of visits in which participants reported HIV preexposure prophylaxis (PrEP) surfing, by prior intention to PrEP surfing (measured on a 7-point Likert scale), in 1217 visits among 475 HIV-negative men who have sex with men with event-driven or no PrEP use, the Amsterdam Cohort Studies, 2021–2023.

Next, we analyzed surfing intention as a predictor for surfing practice in 1217 second or later visits by 475 participants who used event-driven or no PrEP themselves. Higher intention to practice PrEP surfing reported at the previous visit was associated with practicing PrEP surfing in the next six months (Fig. 2, Supplementary Table 1). In multivariable analysis, a higher intention for PrEP surfing was strongly associated with higher odds of subsequently reporting PrEP surfing (aOR 1.6, 95% CI 1.5–1.8, Supplementary Table 1). Thus, each point increase on a 7-point Likert scale increased the odds of practicing PrEP surfing in the next 6 months by 60%. Recent rectal STI or syphilis diagnosis, country of origin other than the Netherlands, having a steady partner in the preceding 6 months and intermediate/high perceived probability of contracting HIV in the coming 6 months were not associated with recent PrEP surfing, but recent PrEP use and number of recent sex partners were (Supplementary Table 1).

## Discussion

PrEP surfing is a common HIV prevention strategy among HIV-negative MSM in and around Amsterdam who do not use PrEP on a daily regimen. Recent PrEP surfing was reported in 25% of all study visits, and 42% of participants reported having practiced PrEP surfing at least once. Participants of younger age, born in the Netherlands, without a steady partner, with 3 or more sex partners, who recently used event-driven PrEP themselves, who had recent rectal STI or infectious syphilis, or who perceived their own probability of contracting HIV as intermediate/high were more likely to have practiced PrEP surfing recently. Moreover, a high intention to practice PrEP surfing was a strong determinant for practicing PrEP surfing in the coming 6 months. Higher intention for PrEP surfing in the coming 6 months was found among those who had CAS with a casual partner, who used PrEP in the preceding 6 months themselves, those without a steady partner, and those with an intermediate/high perceived probability of contracting HIV and those who had intermediate/high level of worrying about contracting HIV in the coming 6 months.

We assessed determinants of recent PrEP surfing and intention to practice PrEP surfing. We found that younger men are more likely to practice PrEP surfing. Another study showed that MSM in Amsterdam discontinuing PrEP use were more often of younger age [21]. The preference to use PrEP surfing might be related to barriers to use PrEP oneself; one might prefer to rely on the PrEP use of a sex partner instead of using it oneself due to barriers. Qualitative studies on reasons for practice PrEP surfing among young MSM are needed to gain insight into underlying motivations. Moreover, we found that MSM who used event-driven PrEP themselves in the preceding 6 months had practiced PrEP surfing more often and had a higher intention to practice it than those who had not used PrEP. MSM who used PrEP themselves are more familiar with PrEP and might feel more secure in assessing and trusting the PrEP use of their sex partner than MSM who did not use PrEP. As a result, MSM who used PrEP might be more inclined to use PrEP surfing as a HIV prevention strategy in a period when they do not use PrEP themselves.

We also found that MSM with an intermediate/high perceived probability of contracting HIV and intermediate/high levels of worrying about contracting HIV in the coming 6 months had a higher intention to practice PrEP surfing. Possible explanations include: these men perceive PrEP surfing as an effective HIV prevention strategy and intend to practice it to prevent themselves from acquiring HIV, considering their perception of high risk for HIV acquisition; or these men intend to practice PrEP surfing and consider it as a less effective preventive strategy, therefore perceive their probability of contracting HIV as higher compared to the use of more effective prevention strategies, such as condoms or using PrEP oneself. Previous qualitative research on PrEP discontinuation supports the first explanation: men discontinued PrEP or perceived their risk on HIV acquisition low when their sex partner was using PrEP [22]. It is important to examine the characteristics and motivations of individuals who rely on PrEP surfing, particularly in terms of risk perception and risk aversion, as this can help to better inform HIV prevention choices during counselling.

We found a strong association between intention for PrEP surfing and the subsequent practice of PrEP surfing. Nevertheless, a non-negligible proportion of participants with lower intention for PrEP surfing practiced it in the following 6 months. One explanation for this might be that for some men, barriers in accessing PrEP themselves could be the reason to engage in PrEP surfing despite initial low intention. Moreover, motives and decision-making can be altered due to contextual factors such as sexualized drug use or social norms of the group that a person belongs to [23,24].

Possibly individuals who face challenges with correct PrEP use or face difficulties in accessing formal PrEP consider PrEP surfing as beneficial strategy. For these individuals, as well as those who do not wish to use PrEP or other prevention methods such as condoms, PrEP surfing may serve as an alternative to reduce HIV acquisition risk compared to using no HIV prevention strategy at all. However, the effectiveness of PrEP surfing in preventing HIV is unknown, and this might differ across different settings. For example, PrEP surfing might not be effective if the partner is not using PrEP as prescribed or has undiagnosed and untreated HIV infection. In addition, effectiveness relies on the reliability of the PrEP use disclosure provided by the sex partner. With known casual partners, reliability might be higher compared to unknown casual partners. Vice versa, having had sex multiple times with the same partner might increase the trust in them, even though the provided information on their PrEP use might not be reliable. On the other hand, PrEP surfing with steady partners might be an effective HIV prevention strategy, as the reliability of a steady partner's information on PrEP use could be very high. However, steady partners do not necessarily always provide reliable information. Therefore, putting trust on reported PrEP use of one's steady partner, might lead to unsafe PrEP surfing practices. In this study, we did not measure the intention to practice PrEP surfing with steady partners. Research into types of partners with whom MSM practice PrEP surfing can provide information on partner types with whom MSM perceive PrEP surfing as a reliable HIV prevention strategy. However, PrEP surfing should not be recommended as HIV-preventive strategy as its effectiveness is dependent on the quality of PrEP use disclosure of the sex partner, and should be strongly discouraged in contexts where uncertainly of a sex partner's PrEP use exists.

Our study has some limitations. First, the ACS consist predominantly of middle-aged MSM with a college-level education, and underrepresents other subpopulations at increased risk of acquiring HIV (e.g. transgender people, people engaging in sex work), limiting generalizability of our results to other populations at risk for HIV acquisition. Second, we did not assess how often MSM practiced PrEP surfing, but only whether they had practiced it in the preceding 6 months. Third, imputation of HIV perception variables might have affected our outcomes. This is unlikely though, since we found that HIV perceptions of individuals did not fluctuate strongly between subsequent visits.

The first strength of this study is that, to our knowledge, it is the first quantitative study assessing PrEP surfing and showed its indisputable presence as HIV-preventive strategy among MSM in and around Amsterdam. Second, we assessed determinants of PrEP surfing, and our results can help to identify MSM who are more likely to practice PrEP surfing (e.g. young MSM without a steady partner) and could be prioritized for counselling. This is useful for professionals, such as general practitioners and healthcare providers in sexual health clinics. Third, due to the longitudinal design of ACS, we were able to show the association of intention to PrEP surfing and actual behavior. Finally, the large sample size of this study enhanced the precision of our estimates.

In conclusion, we established that PrEP surfing is practiced by many HIV-negative MSM who do not use PrEP on a daily regimen in and around Amsterdam, particularly PrEP-experienced younger MSM without a steady partner. Also, MSM with a higher perceived probability of acquiring HIV practice PrEP surfing more often. Research on the effectiveness of PrEP surfing is crucial to optimize counselling on PrEP surfing. Our findings also emphasize the importance of conducting in-depth research regarding the knowledge and assumptions, motivations, decision-making processes, facilitators and barriers of both planning to practice and actually practicing PrEP surfing in order to optimize HIV prevention care.

## Acknowledgements

We would like to thank all study participants and also: Kees de Jong, Ilya Peters and Samantha van den Houten (study nurses), Ertan Ersan and Dominique Loomans (data managers), Anders Boyd (statistician) and Kai Jonas (who introduced the term PrEP surfing).

### Conflicts of interest

All authors declare that they have no conflicts of interest. The Amsterdam Cohort Studies is part of the Netherlands HIV Monitoring Foundation and is financially supported by the Center for Infectious Disease Control of the Netherlands National Institute for Public Health and the Environment, Bilthoven, the Netherlands.

Publication history: An earlier version of this manuscript was posted to SSRN: https://dx.doi.org/10.2139/ssrn.5220658.

## Supplementary Material

Supplemental Digital Content
