# Supplementary material for: PrEP surfing: HIV preexposure prophylaxis use of sex partners as HIV prevention strategy among men who have sex with men
Source: AIDS. 2026 Apr 21;40(9):1365–75. doi: 10.1097/QAD.0000000000004526 (PMC13566479; doi:10.1097/QAD.0000000000004526)
Supplement: Supplementary file 1 [file aids-40-1365-s001.docx]

**Supplementary material to:**

**PrEP surfing: HIV preexposure prophylaxis use of sex partners as HIV prevention strategy among men who have sex with men**

**Authors**

Carien Blomaard^1,2,3,4^, Jeffrey C.D. Koole^1^, Elske Hoornenborg^1,2,3,4^, Maria Prins^1,2,3,4^, Janneke C.M. Heijne^1,2,3,4^, Udi Davidovich^1,5^, Maarten F. Schim van der Loeff^1,2,3,4^

Affiliations:

1. Department of Infectious Diseases, Public Health Service Amsterdam, Amsterdam, The Netherlands
2. Department of Internal Medicine, Amsterdam UMC location University of Amsterdam, Amsterdam, The Netherlands
3. Amsterdam Institute for Immunology and Infectious Diseases (AII), Amsterdam, the Netherlands
4. Amsterdam Public Health Research Institute (APH), Amsterdam, the Netherlands
5. Department of Social Psychology, University of Amsterdam, Amsterdam, The Netherlands

**Supplementary Methods**

Participants were asked to report recently practiced PrEP surfing and intention to PrEP surfing between October 2021 and December 2023. First, they were provided a short explanation of PrEP surfing, and were asked questions about PrEP surfing as follows (please note that at the time we inserted these questions in the questionnaire we used the term “PrEP sorting” rather than “PrEP surfing”, but with identical meaning): *“The following questions are about PrEP sorting. PrEP is a medication that can be taken by HIV-negative individuals to reduce their risk of HIV infection. PrEP can be taken daily or intermittent (around the sex). When you use information about your partner’s PrEP use to decide to have condomless sex with them, we call it PrEP sorting. PrEP sorting can be a way to protect yourself from HIV.*

Then, the following question on recent PrEP surfing was posed:

1. *“Did you use information about your HIV-negative casual partner’s PrEP-use in order to decide to have condomless anal sex with him, in the preceding six months?”*

Answer options: *Yes/no*

Finally, a question on intention to PrEP surfing in the coming 6 months was posed:

2. *“Are you planning to use information about your HIV-negative partner’s PrEP-use in order to decide to have condomless anal sex with him, in the upcoming six months?”*

Answer options: *Definitely not	1	2	3	4	5	6	7	Definitely*

The first question on recent practice of PrEP surfing was only asked to those without HIV reporting steady or casual partners; and the second question to all participants without HIV. Therefore, the number of participants with available data for *recent PrEP surfing* and *intention to practice PrEP surfing* may differ.

**Supplementary Table 1. Assessment of intention-behavior agreement of PrEP surfing in past 6 months in 1,217 study visits among 475 HIV-negative men who have sex with men with event-driven or no PrEP use, participating in the Amsterdam Cohort Studies, the Netherlands, 2021-2023. Results from logistic regression with standard errors adjusting for correlated observations.**

|  |  | Univariable model | | Multivariable model | | |
| --- | --- | --- | --- | --- | --- | --- |
|  | median (IQR)/ n/N (%) | OR | 95% CI | aOR | 95% CI | p |
| Intention to PrEP surfing in preceding visit^a^ | 4 (1-5) | 1.64 | 1.51-1.78 | 1.60 | 1.46-1.75 | <0.001 |
| Age group |  |  |  |  |  |  |
| 16-34 years | 79/248 (31.9%) | REF | - | REF | - | - |
| 35-44 years | 77/241 (32.0%) | 1.00 | 0.63-1.61 | 1.02 | 0.64-1.60 | 0.945 |
| 45-54 years | 81/431 (18.8%) | 0.50 | 0.32-0.77 | 0.71 | 0.46-1.11 | 0.135 |
| ≥55 years | 57/297 (19.2%) | 0.51 | 0.30-0.85 | 0.52 | 0.31-0.86 | 0.011 |
| Country of Origin |  |  |  |  |  |  |
| The Netherlands | 264/1,044 (25.3%) | REF | - |  |  |  |
| Other | 27/164 (16.5%) | 0.58 | 0.35-0.98 |  |  |  |
| Steady partner^b^ |  |  |  |  |  |  |
| Yes | 171/849 (20.1%) | REF | - |  |  |  |
| No | 123/368 (33.4%) | 1.99 | 1.42-2.78 |  |  |  |
| Number of sex partners^b^ |  |  |  |  |  |  |
| 0-2 | 48/513 (9.4%) | REF | - | REF | - | - |
| 3-8 | 126/384 (32.8%) | 4.73 | 3.07-7.30 | 3.90 | 2.41-6.32 | <0.001 |
| ≥9 | 120/320 (37.5%) | 5.81 | 3.66-9.23 | 5.25 | 3.20-8.63 | <0.001 |
| PrEP use^b, c^ |  |  |  |  |  |  |
| No | 93/747 (12.5%) | REF | - | REF |  |  |
| Yes | 201/470 (42.8%) | 5.25 | 3.68-7.51 | 2.57 | 1.78-3.70 | <0.001 |
| Rectal STI or syphilis^b^ |  |  |  |  |  |  |
| No | 242/1,099 (22.0%) | REF | - |  |  |  |
| Yes | 52/118 (44.1%) | 2.79 | 1.83-4.26 |  |  |  |
| Perceived probability of contracting HIV^d^ |  |  |  |  |  |  |
| Low (1-2.5) | 238/1,037 (23.0%) | REF | - |  |  |  |
| Intermediate/high (3-7) | 56/180 (31.1%) | 1.52 | 1.05-2.19 |  |  |  |

Abbreviations: aOR, adjusted odds ratio; CAS, condomless anal sex; CI, confidence interval; HIV, human immunodeficiency virus; IQR, interquartile range; n, number; N, total number; OR, odds ratio; p, p-value; PrEP, oral HIV pre-exposure prophylaxis; STI, sexually transmitted infection

Data were missing for: country of origin (n=9)

^a^Intention to practice PrEP surfing in coming 6 months was measured using a 7-point Likert scale. Interpretation of the adjusted OR of 1.60: with each point increase on intention on the 7-point Likert scale, the odds that a person practiced PrEP surfing in the coming next 6 months increased by 60%

^b^In preceding 6 months

^c^Event-driven or both daily and event-driven PrEP use. Participants using PrEP only on a daily regimen in the preceding 6 months were excluded from this analysis

^d^In coming 6 months

**b. Importance of preventing HIV acquisition, Distribution of values on Likert scale**

**a. Burden of Contracting HIV, Distribution of values on Likert scale**

**c. Worrying about contracting HIV, Distribution of values on Likert scale**

**d. Probability of contracting HIV, Distribution of values on Likert scale**

**Supplementary Figure 1. Distribution of HIV perception variables in 2,187 study visits among 636 men who have sex with men without HIV participating in the Amsterdam Cohort Studies, the Netherlands, 2021-2023.** Because of very skewed distribution of HIV perception variables, for inclusion in univariable and multivariable regression analyses these variables were dichotomized into (a) low/intermediate (≤5) vs high (≥5.5) expected burden of contracting HIV, (b) low/intermediate (≤5) vs high (≥5.5) self-perceived importance of preventing HIV acquisition, (c) low (≤2.5) vs intermediate/high (≥3) worry about contracting HIV and (d) low (≤2.5) vs intermediate/high (≥3) perceived probability of contracting HIV.
